# Efficacy of acupuncture plus pelvic floor muscle training in postpartum urinary incontinence: a systematic review and meta-analysis

**DOI:** 10.3389/fmed.2026.1758659

**Published:** 2026-04-02

**Authors:** Jin-xia Zhang, Yi Yang, Rui Shen, Hui-fang Li

**Affiliations:** 1 Department of Nursing, Tongxiang Maternal and Child Health-Care Center, Tongxiang, Zhejiang, China; 2 Department of TCM Gynecology, Tongxiang Maternal and Child Health-Care Center, Tongxiang, Zhejiang, China; 3 Department of Acupuncture, Tongxiang Maternal and Child Health-Care Center, Tongxiang, Zhejiang, China

**Keywords:** acupuncture, meta-analysis, pelvic floor muscle training, postpartum urinary incontinence, PPUI

## Abstract

**Background:**

Postpartum urinary incontinence (PPUI) significantly impacts women’s quality of life. While pelvic floor muscle training (PFMT) is the standard care, its efficacy can be limited by poor adherence. Acupuncture is increasingly used as an adjunct therapy, but its added value remains controversial due to variability in clinical practice. This systematic review aimed to evaluate the efficacy and safety of acupuncture combined with PFMT for the treatment of PPUI.

**Methods:**

We searched eight databases, including PubMed, Embase, the Cochrane Library, Web of Science, China National Knowledge Infrastructure (CNKI), the Chinese Biomedical Database (CBM), the Chinese Scientific Journals Database (VIP), and Wanfang, from inception to 30 October 2024, for randomized controlled trials (RCTs) comparing acupuncture plus PFMT with PFMT alone. The outcome measures included validated metrics (the 1-h pad test and the International Consultation on Incontinence Questionnaire-Short Form (ICI-Q-SF) score), and the total effective rate. Risk of bias was assessed using the Cochrane risk-of-bias tool, and certainty of evidence was evaluated using the Grading of Recommendations Assessment, Development, and Evaluation (GRADE) approach.

**Results:**

Nine RCTs involving 865 patients were included. For validated metrics, a narrative synthesis of the 1-h pad test and ICI-Q-SF scores showed a trend toward greater improvement in the acupuncture plus PFMT group compared with PFMT alone, although high clinical and statistical heterogeneity (*I*^2^ > 90%) precluded a reliable meta-analysis for these outcomes. Regarding the total effective rate, the pooled analysis showed a significant benefit for the combined therapy (RR = 1.20, 95% CI [1.13, 1.27], *p* < 0.01); however, potential publication bias was observed.

**Conclusion:**

Low- to very-low-certainty evidence suggests that acupuncture as an adjunct to PFMT may offer additional benefits in improving clinical symptoms and quality of life for women with PPUI. However, the high heterogeneity and methodological limitations of the current evidence preclude precise estimation of the effect size for objective outcomes. More rigorous, high-quality RCTs with standardized protocols are required to confirm these findings.

**Systematic review registration:**

https://www.crd.york.ac.uk/PROSPERO/view/CRD42020169815.

## Introduction

Postpartum urinary incontinence (PPUI) is a prevalent complication and a common manifestation of pelvic floor dysfunction (PFD) during the puerperal period (1, 2). The most frequent form of PPUI is stress urinary incontinence (UI) (3, 4). Previous reports indicate that the prevalence of PPUI varies between 3 and 33% (4–7). PPUI is a multifactorial condition (8), influenced by numerous risk factors such as vaginal delivery (9), advanced maternal age (3), multiparity (10), prenatal obesity (7), urinary incontinence during pregnancy (11, 12), high neonatal birth weight (13), constipation (14), family history of PFD (15), and perineal laceration (13). Epidemiological data further suggest that pregnancy and childbirth are independent risk factors for PPUI (12, 16). Pregnancy and vaginal delivery increase intra-abdominal pressure and levator ani muscle tension, thereby contributing to impaired pelvic floor support. These changes can ultimately lead to the development of urinary incontinence, pelvic organ prolapse, and anal incontinence (17). PPUI may persist into later life (18), affecting not only a woman’s physical wellbeing but also her psychological and socioeconomic status (9, 19). Women with PPUI often report the lowest health-related quality of life (19). Given the high prevalence and potentially severe consequences of PPUI, developing effective treatment strategies is essential for those affected by this condition. Currently, the treatment of PPUI primarily involves non-surgical therapies, such as pelvic floor muscle training (PFMT), behavioral therapy, electrostimulation, and medication (20–23). Among these therapies, PFMT, also known as Kegel exercises, is currently recognized as the first-line treatment for the prevention and management of PPUI, with moderate- to high-quality evidence, regardless of the type of incontinence (24, 25). However, current studies have shown that the effectiveness of PFMT depends on feasibility and maternal adherence, which are often problematic (26–28).

The majority of patients with PPUI have turned to alternative therapies, particularly traditional Chinese medicine (TCM), including acupuncture, moxibustion, and herbal treatments. These therapies can be used alone or in combination with other treatments to reduce adverse reactions and achieve better outcomes. Acupuncture has gained significant attention in recent years due to its simple administration, minimal side effects, low economic burden, and effective therapeutic outcomes. Several systematic reviews have been published on the effectiveness of acupuncture in treating urinary incontinence (UI) (29, 30), and several studies have explored its efficacy in treating PPUI (31, 32). Beyond traditional theories, modern biomedical research suggests that acupuncture, particularly at lumbosacral points, functions as a form of neuromodulation. It is hypothesized to stimulate the S2–S4 sacral nerve roots, thereby modulating the micturition reflex and enhancing the contractility of pelvic floor muscles through the pudendal nerve (33, 34). However, there is currently no systematic review specifically focused on PPUI. In this context, we conducted a systematic review and meta-analysis of published studies to evaluate the efficacy of combining acupuncture with PFMT in women with PPUI.

## Materials and methods

### Methods

This systematic review and meta-analysis was conducted in accordance with the Preferred Reporting Items for Systematic Reviews and Meta-Analyses (PRISMA) guidelines (35). The registration number in the International Prospective Register of Systematic Reviews (PROSPERO) is CRD42020169815.

### Types of studies

This review included randomized controlled trials (RCTs) that evaluated the use of acupuncture for managing PPUI, with no restrictions on publication status or language. Non-RCTs, case reports, conference abstracts, and animal studies were excluded.

### Types of participants

Only female participants who met at least one of the current clinical diagnostic criteria for PPUI were included, regardless of ethnicity, nationality, education, or economic status. All of the participants should have received acupuncture treatment within 1 year postpartum.

### Types of interventions

Patients who had undergone acupuncture treatment combined with PFMT were included as the experimental group, with no limitation on acupuncture types, frequency, or course of treatment. Trials in which patients received other stimulation techniques, such as acupressure, moxibustion, acupoint injection, laser acupuncture, or cupping, were excluded. The control group included patients treated only with PFMT.

### Types of outcome measures

The outcome measures included (1) validated clinical and objective metrics, specifically the 1-h pad test and the International Consultation on Incontinence Questionnaire–Short Form (ICI-Q-SF), and (2) the clinical response rate, measured as the total effective rate. The total effective rate was determined based on the clinical symptom improvement criteria in each study, typically categorized as cured, markedly effective, or effective. The specific criteria for effectiveness varied slightly across the included studies (based on the percentage reduction in leakage or symptom scores). Detailed definitions for each study are summarized in Supplementary Table S1.

### Search strategy

Clinical studies were retrieved from several databases, including PubMed, Embase, the Cochrane Library, Web of Science, China National Knowledge Infrastructure (CNKI), the Chinese Biomedical Database (CBM), the Chinese Scientific Journals Database (VIP), and the Wanfang Database. There were no limitations on language or publication status. The search focused on RCTs examining the effects of acupuncture in the treatment of PPUI, covering the period from the inception of each database to 30 October 2024. We constructed the retrieval strategy based on the PICOS framework. When working with Chinese academic databases, such as CNKI, the following formula was typically used for retrieval: SU = (acupuncture + electroacupuncture + warm acupuncture + needle + needle stimulation) AND (puerperal urinary incontinence + postnatal urinary incontinence + postpartum urinary incontinence + PPUI) AND (randomization + randomized controlled + random grouping + RCT + clinical research). For other databases, PubMed served as an example, and the corresponding search strategy can be found in the Supplementary Table. The search terms were carefully translated for use in other databases. Furthermore, we conducted a manual review of the references from both original and review articles to identify relevant studies.

### Data extraction and quality assessment

Two authors (YY and RS) independently extracted the data using a pre-established and standardized form, including first author, year of publication, sample size, age, treatment interventions and control groups, treatment duration, and outcomes. Any discrepancies were addressed and resolved by a third author (HFL). The methodological quality of each study was independently assessed by the two researchers (JXZ and YY) following the guidelines outlined in the Cochrane Handbook for Systematic Reviews of Interventions. The evaluation was based on the following criteria: random sequence generation, allocation concealment, blinding of participants and personnel, blinding of outcome assessment, incomplete outcome data, selective reporting, and other biases. Each study was classified as having a low, high, or unclear risk of bias. In case of any disagreements, the views of a third researcher (HFL) were consulted to resolve the issue.

### GRADE evaluation

The quality of outcomes was assessed using the Grading of Recommendations Assessment, Development, and Evaluation (GRADE) system, based on the following criteria: study design, risk of bias, inconsistency of results, indirectness of evidence, and other factors. The overall quality of evidence was categorized as high, moderate, low, or very low.

### Statistical analysis

This meta-analysis was conducted using Review Manager (RevMan) software (version 5.3, Copenhagen: The Nordic Cochrane Centre, The Cochrane Collaboration, 2014) and Stata (version 12.0, Stata Corp LP, USA). For the study outcomes, relative risk (RR) with a 95% confidence interval (CI) was used for binary variables, while the weighted mean difference (WMD) with a 95% CI was used for continuous variables. Heterogeneity among the studies was assessed using Cochrane’s *p*-values and the *I*^2^ statistic. To explore potential sources of heterogeneity, subgroup analyses were pre-specified based on the type of acupuncture intervention (electroacupuncture vs. manual/warm acupuncture). Sensitivity analyses were also performed by sequentially omitting individual studies to assess the robustness of the pooled results. Given the inherent clinical and methodological diversity across the included studies—particularly regarding acupuncture modalities (manual vs. electroacupuncture), treatment frequencies, and patient baseline characteristics—we anticipated underlying true effect differences. Therefore, a random-effects model was chosen as the most appropriate and conservative approach to account for this expected variance, even in cases where statistical heterogeneity (*I*^2^) appeared low. However, when substantial clinical and statistical heterogeneity (*I*^2^ > 75%) was identified and could not be sufficiently explained by subgroup analyses, quantitative synthesis (data pooling) was not performed. Instead, a narrative synthesis was conducted to describe the direction and magnitude of the treatment effects for these outcomes. Funnel plots and Begg’s test were used to assess potential publication bias.

## Results

### Search results

**Figure 1:**
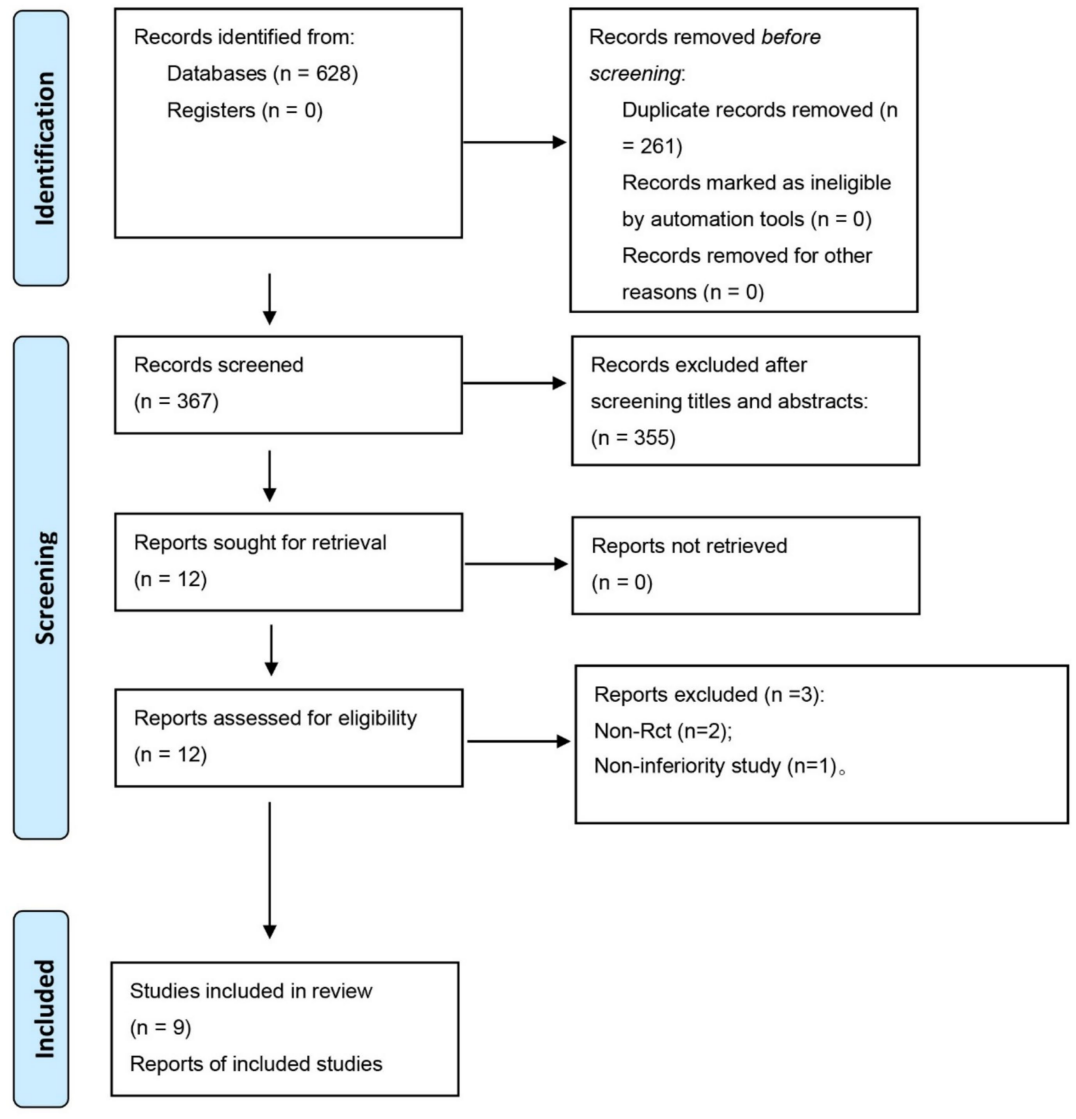
Flow diagram of the study selection process.

As shown in Figure 1, a total of 628 potentially relevant studies examining the effect of acupuncture for PPUI were initially identified. After removing duplicate studies and screening the titles and abstracts, 616 studies were excluded. Subsequently, 12 studies that were deemed potentially eligible were selected for further evaluation. After a thorough assessment, three trials were excluded for various reasons: one study was not an RCT, one study involved mixed interventions, and one study did not report the listed outcomes. Ultimately, nine prospective trials were included in the final meta-analysis (36–44). A thorough manual examination of the reference lists in these studies revealed no additional eligible studies.

### Study characteristics

Table 1 presents a summary of the essential information extracted from the included RCTs. All included studies were conducted and published in China. The studies collectively involved 865 patients diagnosed with PPUI, with 433 participants assigned to the treatment group and 432 to the control group. The sample sizes varied between 61 and 124. No notable differences were found in the baseline characteristics of the participants across the trials. Among these trials, eight used a two-arm design and one used a three-arm design (39).

**Table 1:** Detailed information on the included studies.

Studies	Sample size (n)		Age (y)		Intervention measures		Duration treatment (weeks)	Main outcomes
	T	C	T	C	T	C		
Zhou (44)	49	49	28.34 ± 2.79	27.28 ± 2.68	Electroacupuncture+PFMT 1. Acupoints: Four sacral needles. 2. A depth of 75 to 110 mm. 3. 20–30 min at 2.0 Hz. 4. 1 time/day for 4 weeks.	PFMT	4	①②
Huang (42)	45	45	32.7 ± 7.0	31.9 ± 6.8	Electroacupuncture+PFMT 1. Acupoints: DU20, RN3, RN4, RN6, SP9, ST36, and SP6. 2. A depth of 16.67–33.33 mm. 3. 30 min at 50 Hz, 1–5 mA. 4. 3 times/week for 4 weeks.	PFMT	4	①③
Cao (40)	31	30	27.32 ± 2.93.41	28.16 ± 3.20	Warm acupuncture+PFMT 1. Acupoints: RN6, RN4, RN3, BL23, and BL28. 2. 5 times/week for 8 weeks.	PFMT	8	①②
Yang (36)	38	38	30.55 ± 3.35	30.95 ± 3.45	Acupuncture+PFMT 1. Acupoints: RN3, KI3, LU5, SP6, RN4, RN6, ST36, BL23, and EX-CA1. 2. 5 times/week for 8 weeks.	PFMT	8	①③
Ma (37)	62	62	28.63 ± 1.17	29.15 ± 1.23	Electroacupuncture+PFMT 1. Acupoints: RN6, EX-CA1, ST36, RN3, and SP6. 2. A depth of 16.67–33.33 mm. 3. 30 min at 15 Hz, 5 mA. 4. 3 times/week for 4 weeks.	PFMT	8	①②③
Yan (41)	50	50	32.7 ± 7.0	30.8 ± 6.9	Acupuncture+PFMT 1. Acupoints: EX-CA1, RN4, RN6, DU20, GB34, LU9, KI3, ST36, and SP6. 2. 5 times/week for 4 weeks.	PFMT	4	①③
Zhang (38)	48	48	32.27 ± 6.82	31.84 ± 6.57	Warm acupuncture+PFMT 1. Acupoints: BL32, BL35, BL23, and ST36. 2. 5 times/week for 8 weeks.	PFMT	8	①③
Sun (39)	60	60	30.25 ± 1.84	30.85 ± 1.64	Acupuncture+PFMT 1. Acupoints: EX-CA1, RN4, DU20, ST36, and SP6. 2. 2 time/week for 24 weeks.	PFMT	3	①
Wang (43)	50	50	35.65 ± 4.41	35.68 ± 4.48	Electroacupuncture+PFMT 1. Acupoints: RN4, RN3, ST36, RN6, SP6, BL23, BL20, BL28, BL32, and BL33. 2. A depth of 20–50 mm. 3. 30 min at 2/10 Hz, 0.1–1.0 mA. 4. 6 time/week for 12 weeks.	PFMT	12	①②③

T, trial group; C, control group; ES, electrical stimulation; PFMT, pelvic floor muscle training. ①: the total effective rate; ②: 1-h pad test; ③: ICIQ-SF score.

### Risk of bias assessment

**Figure 2:**
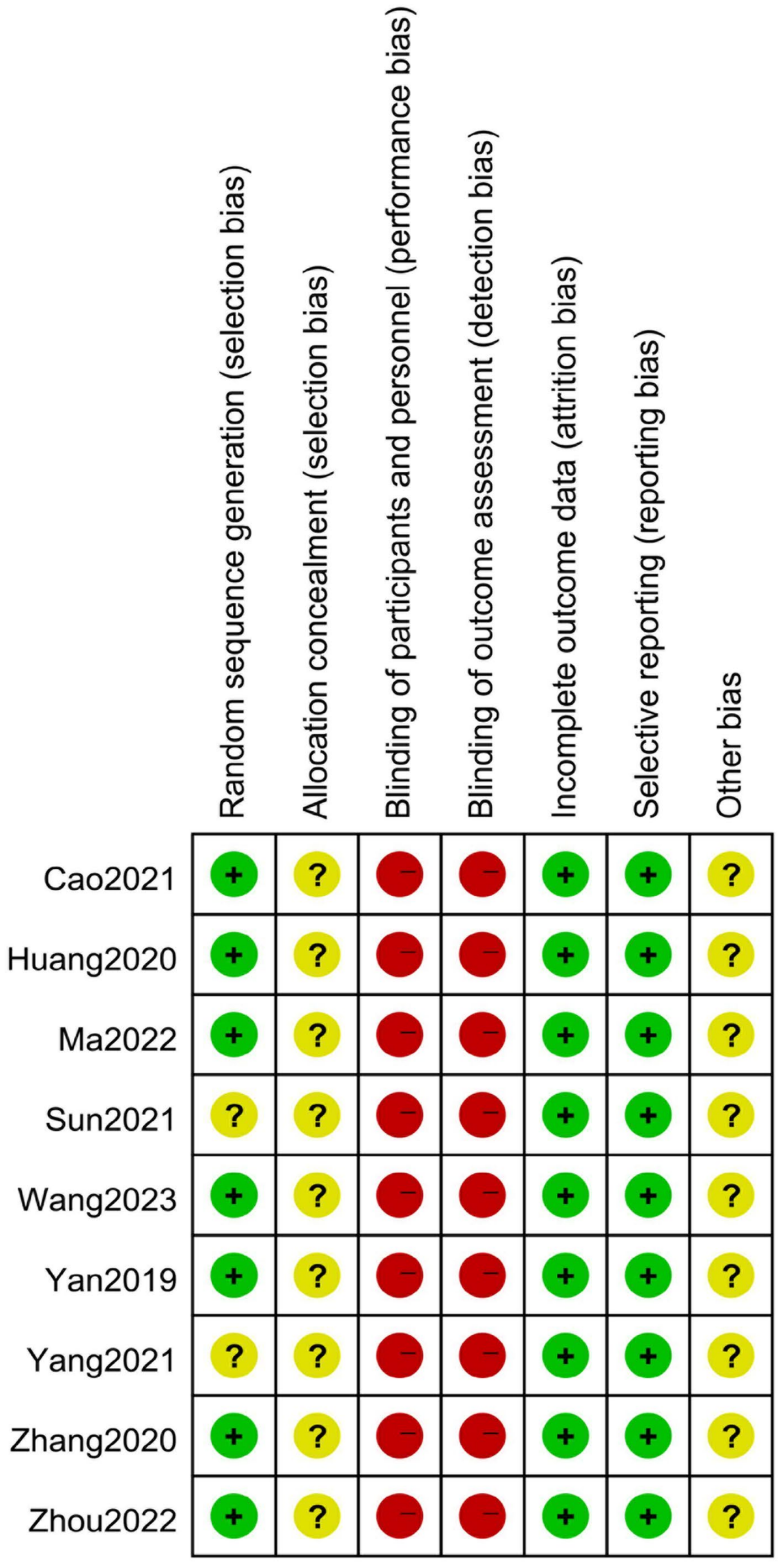
Risk bias assessment of all included studies.

Overall, the methodological quality of the trials was assessed as poor. Among them, seven trials reported the use of appropriate randomization methods, such as random number tables or coin tosses, which were associated with a low risk of bias (37, 38, 40–44). Two of the trials did not provide a clear description of the randomization procedure (36, 39). No trials reported details on allocation concealment or blinding of either patients or investigators. Additionally, none of the studies reported the number or reasons for dropouts. There was no evidence of selective reporting in any of the trials. Furthermore, none of the included studies calculated the sample size. The findings from the assessments are presented in Figure 2.

### Outcome measures

**Figure 3:**
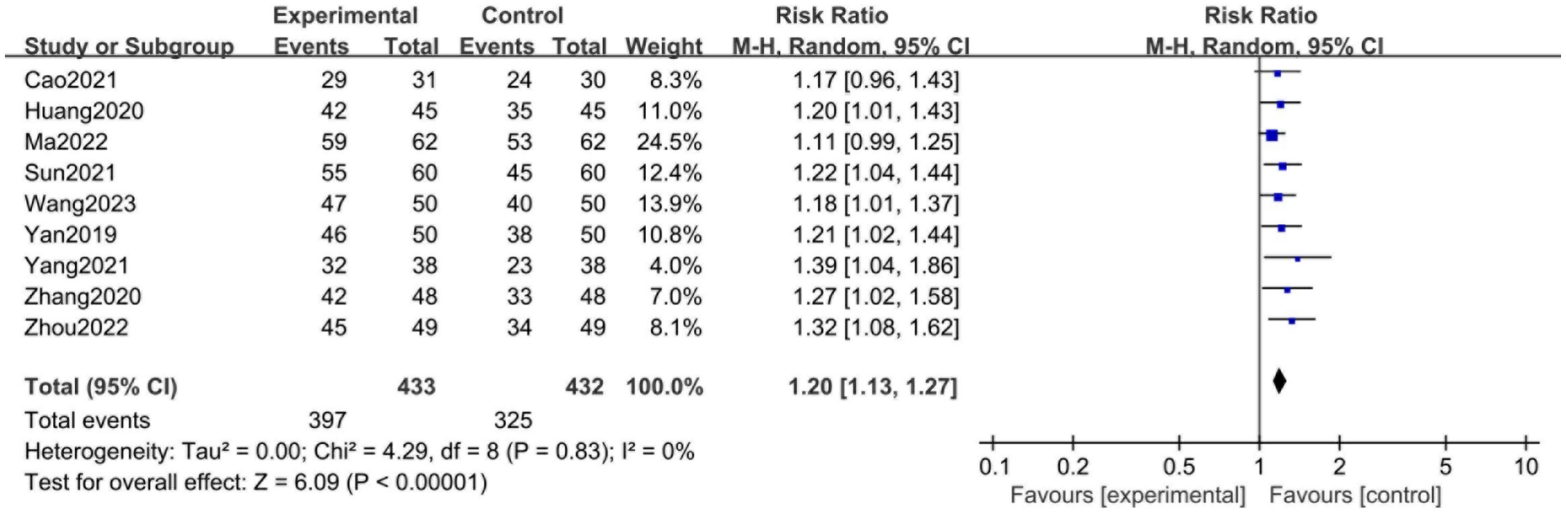
Forest plot of the overall effective rate.

All nine studies (36–44) reported the total effective rate. There was no significant heterogeneity among the studies (*I*^2^ = 0%, *p* = 0.83). The random-effects model showed that the total effective rate in the observation group (acupuncture + PFMT) was significantly higher than that in the control group (PFMT alone) (RR = 1.20, 95% CI [1.13, 1.27], *p* < 0.01, *I^2^* = 0%, Figure 3). This result indicates a consistent clinical benefit of the combination therapy.

**Figure 4:**
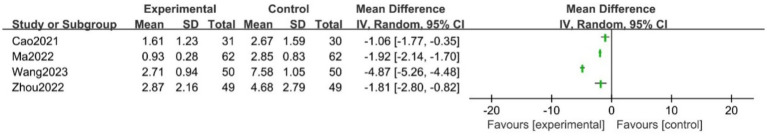
Forest plot of the 1-h pad test.

Four studies (37, 40, 43, 44) reported the urine leakage weight using the 1-h pad test. Due to substantial clinical and statistical heterogeneity (*I*^2^ = 98%) that could not be explained using a subgroup analysis, we did not pool the data for the meta-analysis. Instead, a narrative synthesis was performed. As shown in Figure 4, all four studies consistently reported a significant reduction in urine leakage in the acupuncture group compared with the control group.

**Figure 5:**
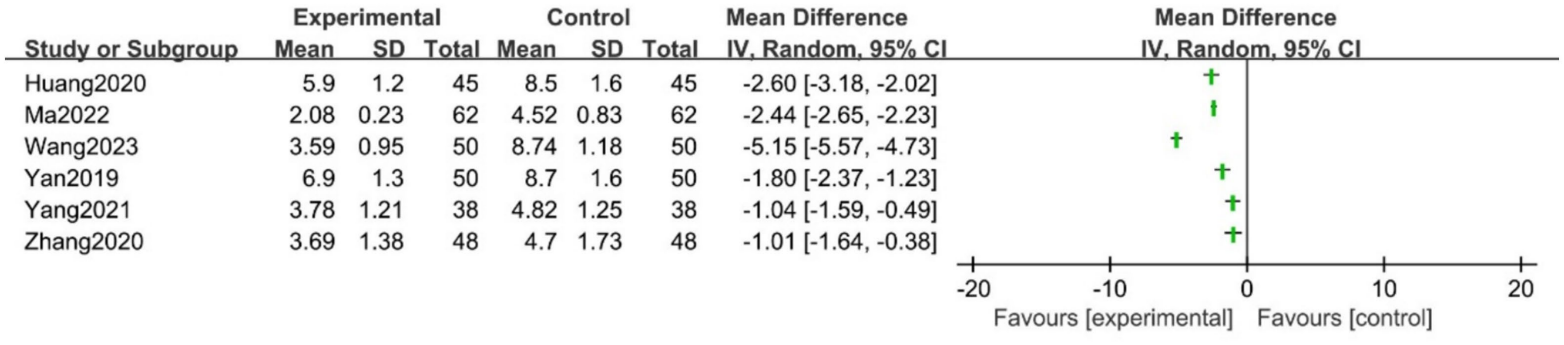
Forest plot of the ICI-Q-SF score (ICI-Q-SF, Incontinence Questionnaire–Short Form).

Five studies evaluated quality of life using the ICI-Q-SF score. Similar to the pad test, extremely high heterogeneity (*I*^2^ = 97%) could not be explained by subgroup analysis, which precluded quantitative pooling. Figure 5 illustrates the individual effect estimates for each study. A descriptive analysis showed that all five trials reported statistically significant lower scores in the acupuncture group compared with the control group.

An evaluation of adverse events was reported in only two studies. Wang (43) explicitly stated that no treatment-related adverse events occurred in either the acupuncture group or the control group. Ma (37) stated that the treatment was safe, with no toxic side effects. The remaining seven studies did not explicitly report safety data or adverse events.

### Publication bias and sensitivity analysis

**Figure 6:**
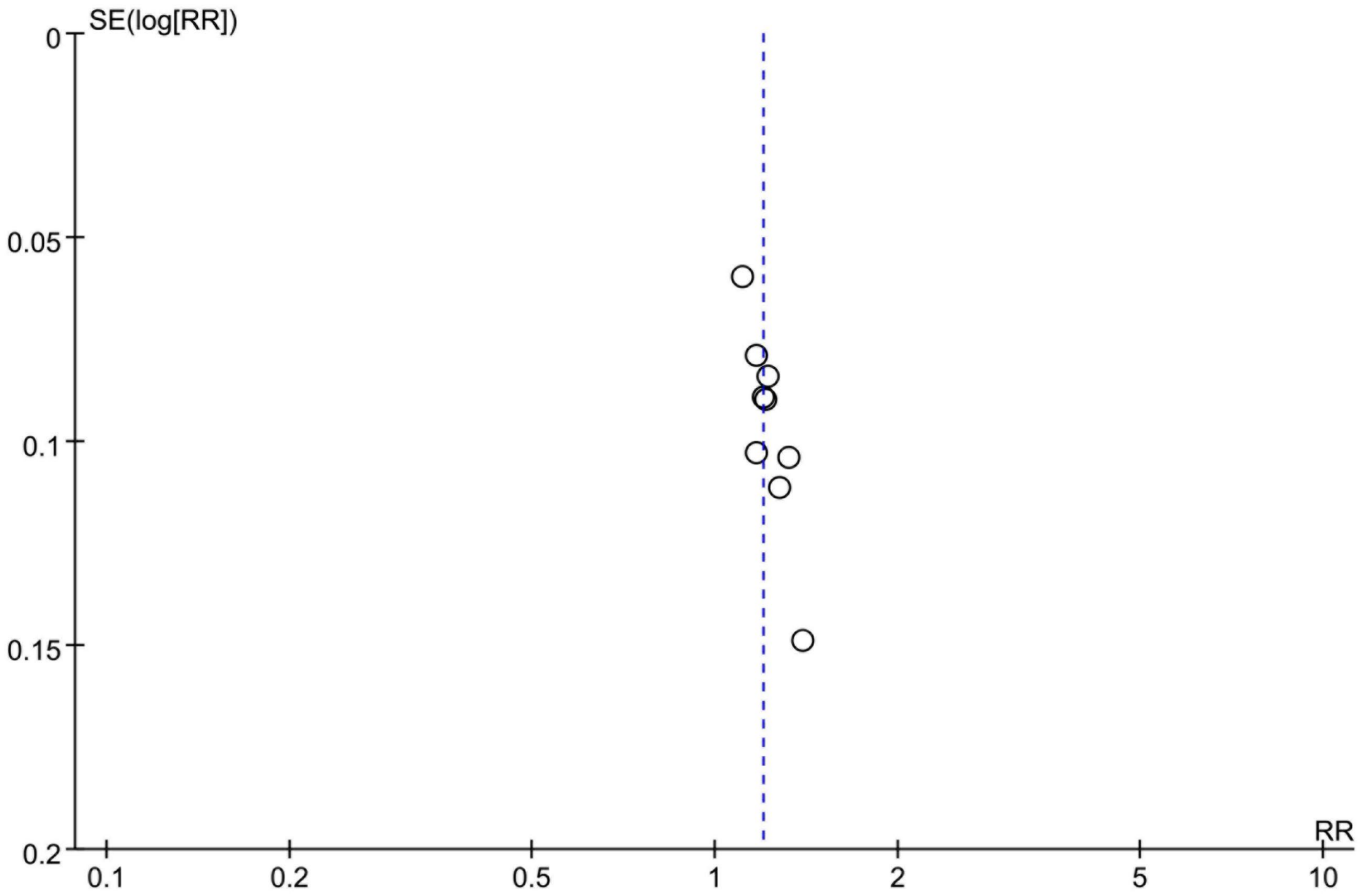
Funnel plot of the overall effective rate.

Visual inspection of the funnel plot for the total effective rate revealed some asymmetry (Figure 6), which was statistically confirmed by Begg’s test (*p* = < 0.05), indicating a significant presence of publication bias.

**Figure 7:**
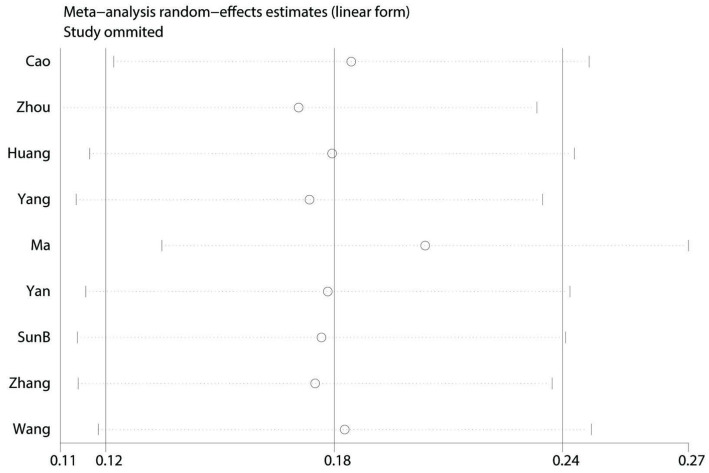
Sensitivity analysis of the overall effective rate.

Despite this, a sensitivity analysis was performed for the total effective rate by sequentially omitting individual studies. The pooled effect estimate remained stable and statistically significant throughout the process, indicating the robustness of the total effective rate (Figure 7). Notably, sensitivity analyses were not performed for other outcomes (1-h pad test and ICI-Q-SF) because quantitative data pooling was not conducted due to high heterogeneity.

### GRADE results

According to the GRADE system, the certainty of the evidence ranged from low to very low (Table 2). The overall quality of evidence was downgraded primarily due to a serious risk of bias and suspected publication bias. Additionally, serious inconsistency was identified for other outcomes (1-h pad test and ICI-Q-SF) due to substantial unexplained heterogeneity (*I*^2^ > 90%).

**Table 2:** Summary of GRADE.

Outcome	Included studies (*n*)	Patients (*n*)	Quality of evidence	Reasons
Overall effective rate	9	865	⨁⨁◯◯ LOW	“Risk of bias” and “other factors” were downgraded to “serious.”
1-h pad test	4	383	⨁◯◯◯ VERY LOW	“Risk of bias,” “inconsistency,” and “other factors “were downgraded to “serious.”
ICI-Q-SF score	6	586	⨁◯◯◯ VERY LOW	“Risk of bias,” “inconsistency,” and “other factors “were downgraded to “serious.”

GRADE, Grading of Recommendations Assessment, Development, and Evaluation; ICI-Q-SF, Incontinence Questionnaire–Short Form.

## Discussion

In this systematic review and meta-analysis of 9 RCTs involving 865 patients, we evaluated the added value of acupuncture to PFMT for the treatment of PPUI. The results suggested that combining acupuncture with PFMT may provide an additive effect in managing PPUI. While the total effective rate showed a statistically significant improvement, this result must be interpreted with caution, as it is derived from trials with non-standardized outcome reporting and low-certainty evidence.

In TCM theory, PPUI is primarily related to the deficiency of kidney qi and unfavorable gasification of the bladder, which makes the normal method of urination difficult (45, 46). It can also result from the weakness of postpartum women due to insufficiency of the lungs and spleen qi, which may lead to an inability to control urine (47). Acupuncture, a widely used TCM therapy, has been extensively applied by clinicians in China and other countries as a complementary and alternative therapy for managing a variety of diseases and promoting overall health recovery (48–51). According to some studies, in the treatment of PPUI, acupuncture can strengthen kidney qi, support the recovery of bladder function, and improve quality of life with few adverse events (33, 52, 53). Previous studies have also found that acupuncture can enhance urinary control, alleviate symptoms of urinary incontinence, and provide more effective long-term outcomes for patients with PPUI (54). Many lumbosacral acupuncture points referenced in the literature included in those studies are situated in the same or nearby spinal segments as the nerves that innervate the bladder. These acupuncture points can further influence the urination function by modulating the sacral pulp urination center, finally achieving therapeutic effects (25).

The superior efficacy of the combination therapy can be explained by the synergistic mechanisms of sacral neuromodulation and pelvic floor muscle recovery. Biomedically, acupuncture stimulation at lumbosacral points is hypothesized to modulate the micturition reflex (55). Anatomically, these acupoints correspond to the S2–S4 sacral nerve roots. Stimulation of these segments can activate the pudendal nerve, enhance the contractility of the external urethral sphincter, and inhibit the parasympathetic discharge to the detrusor muscle, thereby improving urinary control (33). Furthermore, electroacupuncture has been shown to promote the regeneration of collagen fibers in pelvic floor tissues, offering a physiological basis for the observed improvements in objective leakage outcomes (56).

A key observation in this review was the high statistical heterogeneity in the 1-h pad test and the ICI-Q-SF, which persisted across the subgroup analysis. This heterogeneity likely reflects the methodological diversity in current clinical practice. For instance, the protocols for the 1-h pad test varied significantly regarding pre-test water intake and exercise intensity across different hospitals. Similarly, the ICI-Q-SF is a subjective reporting tool susceptible to individual perception bias. Despite this variation in effect magnitude, the direction of effect remained consistent across all studies, supporting the robustness of the conclusion that acupuncture provides added benefit.

This study was the first-ever meta-analysis estimating acupuncture in the treatment of PPUI. The study design adheres to all relevant guidelines for systematic reviews and meta-analyses. Despite our comprehensive analysis and evaluation of all included studies, there are several limitations that must be acknowledged. First, although the total effective rate is widely used in Chinese clinical trials, it lacks a standardized international definition. Consequently, although the pooled result showed statistical significance, the clinical interpretability of this composite endpoint should be viewed with caution compared with objective measures such as the 1-h pad test. Second, due to issues such as unclear allocation concealment, selective bias, and inconsistent blinding methods, the quality of the studies was low. Third, although we conducted an unbiased literature search with no language restrictions, all of the trials included were conducted in China and published in Chinese. Fourth, few studies reported adverse reactions during or after treatment, and none provided long-term follow-up data, leaving the long-term effects of the intervention unclear.

## Conclusion

Current evidence from this systematic review suggests that acupuncture, as an adjunctive therapy to PFMT, may offer potential benefits in improving clinical symptoms and quality of life for women with PPUI. However, these findings should be interpreted with caution due to the high risk of performance bias, the subjective nature of the clinical effective rate, and substantial heterogeneity across studies. The current evidence is graded as low to very low certainty. Therefore, while acupuncture shows potential, its safety profile requires further validation through trials with standardized adverse event reporting.

## Data Availability

The original contributions presented in the study are included in the article/Supplementary material further inquiries can be directed to the corresponding author.
