# Supplementary material for: Efficacy of acupuncture plus pelvic floor muscle training in postpartum urinary incontinence: a systematic review and meta-analysis
Source: Front Med (Lausanne). 2026 Apr 2;13:1758659. doi: 10.3389/fmed.2026.1758659 (PMC13083168; doi:10.3389/fmed.2026.1758659)
Supplement: Supplementary file 1 [file Table_1.docx]

Supplementary Table S1. The definitions of total effective rate in studies.

| **Study ID** | **Categories** | **Detailed Criteria for Efficacy** | **Calculation of Total Effective Rate** |
| --- | --- | --- | --- |
| **Zhou (2022)** | 3 Levels | **Markedly effective :** Leakage reduction >70%.  **Effective:** Leakage reduction > 50%.  **Ineffective:** Leakage reduction < 50% or increased. | (Markedly + Effective) / Total |
| **Huang (2020)** | 4 Levels | **Cure:** Symptoms completely disappeared.  **Markedly effective:** Pad usage reduction > 50%.  **Effective:** Pad usage reduction < 50%.  **Ineffective:** No improvement or aggravated. | (Cure + Markedly + Effective) / Total |
| **Ma (2022)** | 4 Levels | **Cure:** Symptoms disappeared, no leakage.  **Effective:** Leakage reduction >75%.  **Markedly effective:** Leakage reduction 25%–74%.  **Ineffective:** Leakage reduction < 25%. | (Cure + Markedly + Effective) / Total |
| **Wang (2023)** | 3 Levels | **Cure:** No leakage on exertion, voluntary control.  **Effective:** Leakage frequency reduced, occasional leakage.  **Ineffective:** Leakage persists with life impact. | (Cure + Effective) / Total |
| **Yang (2021)** | 3 Levels | **Markedly effective:** Can control urination, no leakage on exertion.  **Effective:** Leakage amount reduced on exertion.  **Ineffective:** No improvement. | (Markedly + Effective) / Total |
| **Cao (2021)** | 3 Levels | **Cure:** Symptoms disappeared; Pad test negative.  **Effective:** Leakage frequency reduction > 50%.  **Ineffective:** Leakage frequency not reduced. | (Cure + Effective) / Total |
| **Zhang (2020)** | 4 Levels | **Cure:** Symptoms completely disappeared.  **Markedly effective:** Leakage reduction > 50%.  **Effective :** Leakage reduction 20%–50%.  **Ineffective:** No improvement. | (Cure + Markedly + Effective) / Total |
| **Yan (2019)** | 4 Levels | Categories listed as Cure, Markedly effective, Effective, and Ineffective. Quantitative criteria were not explicitly detailed in the methods section. | (Cure + Markedly + Effective) / Total |
| **Sun (2021)** | 3 Levels | **Cure:** No leakage on exertion (maintained for 3 months).  **Markedly effective:** 1-hour Pad test < 2g.  **Ineffective:** 1-hour Pad test > 2g. | (Cure + Markedly) / Total |
