# Supplementary material for: Efficacy of acupuncture plus pelvic floor muscle training in postpartum urinary incontinence: a systematic review and meta-analysis
Source: Front Med (Lausanne). 2026 Apr 2;13:1758659. doi: 10.3389/fmed.2026.1758659 (PMC13083168; doi:10.3389/fmed.2026.1758659)
Supplement: Supplementary file 2 [file Table_2.docx]

| **Supplement Table Search strategy used in PubMed database** | |
| --- | --- |
| #1 | Acupuncture therapy[MeSH Terms] |
| #2 | Acupuncture[MeSH Terms] |
| #3 | Acupuncture Treatment[Title/Abstract] |
| #4 | Acupuncture∗[Title/Abstract] |
| #5 | Warm needling[Title/Abstract] |
| #6 | Body acupuncture[Title/Abstract] |
| #7 | Fire needling[Title/Abstract] |
| #8 | Manual acupuncture[Title/Abstract] |
| #9 | Electroacupuncture[Title/Abstract] |
| #10 | Scalp acupuncture[Title/Abstract] |
| #11 | Auricular acupuncture[Title/Abstract] |
| #12 | Dermal needle[Title/Abstract] |
| #13 | Elongated needle[Title/Abstract] |
| #14 | Plum blossom needle[Title/Abstract] |
| #15 | Ear acupuncture[Title/Abstract] |
| #16 | #1 OR to #15 |
| #17 | Postpartum Thyroiditides [MeSH Terms] |
| #18 | Postpartum Period[MeSH Terms] |
| #19 | Postpartum[Title/Abstract] |
| #20 | Puerperium[Title/Abstract] |
| #21 | Post partum[Title/Abstract] |
| #22 | Pregnancy[MeSH Terms] |
| #23 | #17 OR to #22 |
| #24 | Urinary Incontinence[MeSH Terms] |
| #25 | Urinary Incontinence, Urge[MeSH Terms] |
| #26 | Urinary Incontinence, Stress[MeSH Terms] |
| #27 | #24 OR to #26 |
| #28 | #23 and #27 |
| #29 | randomized controlled trial[Publication Type] |
| #30 | controlled clinical trial[Publication Type] |
| #31 | randomized[Title/Abstract] |
| #32 | randomly[Title/Abstract] |
| #33 | placebo[Title/Abstract] |
| #34 | trial[Titlel] |
| #35 | #29 OR to #34 |
| #36 | #16 AND #28 AND #35 |
